# New Sulphated Flavonoids and Larvicidal Activity of *Helicteres velutina* K. Schum (Sterculiaceae)

**DOI:** 10.3390/molecules23112784

**Published:** 2018-10-27

**Authors:** Diégina A. Fernandes, Maria S. R. Souza, Yanna C. F. Teles, Louise H. G. Oliveira, Jéssica B. Lima, Adilva S. Conceição, Fabíola C. Nunes, Tania M. S. Silva, Maria de Fátima Vanderlei de Souza

**Affiliations:** 1 Post Graduation Program in Bioactive Natural and Synthetic Products, Federal University of Paraíba, João Pessoa 58051-900, PB, Brazil; diegina@ltf.ufpb.br (D.A.F.); sallett@ltf.ufpb.br (M.S.R.S.); 2 Department of Chemistry and Physics, Agrarian Sciences Center, Federal University of Paraíba, Areia 58397-000, PB, Brazil; yanna@cca.ufpb.br; 3 Biotechnology Center, Federal University of Paraíba, João Pessoa 58051-900, PB, Brazil; louiseguimaraes@outlook.com (L.H.G.O.); fabiola@cbiotec.ufpb.br (F.C.N.); 4 Post Graduation Program in Plant Biodiversity, Department of Education, University of the State of Bahia, Paulo Afonso 41150-000, BA, Brazil; jessica.bl@hotmail.com (J.B.L.); adilva.souza@gmail.com (A.S.C.); 5 Department of Molecular Sciences, Rural Federal University of Pernambuco, Campus Dois Irmãos, Recife 52171-900, PE, Brazil; sarmentosilva@gmail.com; 6 Post Graduation in Development and Technological Innovation in Medicines, Federal University of Paraíba, João Pessoa 58051-900, PB, Brazil

**Keywords:** *Helicteres velutina*, *Sterculiacaeae*, sulphated flavonoids, larvicidal activity, *Aedes aegypti*

## Abstract

*Helicteres velutina* K. Schum (Sterculiaceae), commonly known in Brazil as ‘pitó’, is traditionally used by indigenous peoples as insecticides and repellents. The present work reports on the the phytoconstituents from aerial parts of *H. velutina* and evaluation of the larvicidal potential of its extract. The compounds were isolated using chromatographic techniques and identified by NMR, IR and LC-HRMS. This study led to the isolation of a fatty acid, one aliphatic alcohol, four chlorophyll derivatives, one steroid, triterpenes, a lignan, and flavonoids, highlighting the new compounds in the literature, 5,4′-di-hydroxy-7-methoxy-8-*O*-sulphate flavone (mariahine) (**15a**) and 5,3′-di-hydroxy-7,4′-dimethoxy-8-*O*-sulphate flavone (condadine) (**15b**). The work presented here contributes to the chemotaxonomic knowledge of the Sterculiaceae family by describing the occurrence of sulphated flavonoids in this family for the first time. The crude ethanolic extract of *H. velutina* featured robust larvicidal activity against *Aedes aegypti* larvae, showing that the extract can be useful as a domestic larvicide, just as indicated by traditional use, to combat *A. aegypti*, a vector insect of severe viral diseases, such as dengue and Zika.

## 1. Introduction

The Sterculiaceae family is composed of 70 genera and approximately 1500 species spread all over the world, with 14 genera and 165 species found in Brazil [1,2]. According to the system, Angiosperm Phylogeny Group (APG) IV, the families Sterculiaceae, Bombacaceae and Tiliaceae were insert into the family Malvaceae sensu lato [3]. The *Helicteres* genus has a predominantly pantropical distribution in American and Asian countries. There is no record of the species occurring in both continents [4]. Phytochemical studies on *Helicteres* species have demonstrated the presence of terpenes [5], steroids [6], alkaloids [7], saponins [8] and flavonoids [9]. Many *Helicteres* species are traditionally used as medicines, for example, *Helicteres sacarolha* is used to treat hypertension and ulcers [9] and *Helicteres angustifólia* has been employed as an analgesic and anti-inflammatory herb [6]. The traditional uses of *Helicteres* species have raised scientific interest in their pharmacological activities. *Helicteres isora* has been shown to possess antioxidant, antimicrobial and hepatoprotective activities [8], and *H. angustifólia* was active against the hepatitis B virus [10].

*Helicteres velutina*, popularly known as ‘pitó’, is an endemic species from Brazil. The plant is traditionally used as an insect repellent by indigenous peoples from the Pankararé tribe in Paulo Afonso, Bahia [11]. There are literature reports of the biological activities of roots and stem extracts against the larvae of *Aedes aegypti* [11]. However, no subsequent biological studies were carried out and no phytochemical studies are available.

Considering the lack of phytochemical information and the interesting uses of *H. velutina*, this work aims to isolate and identify compounds from their aerial parts and evaluate the larvicidal activity of the obtained extract against larvae of *A. aegypti*.

## 2. Results

### 2.1. Identification of Compounds

**Figure 1:**
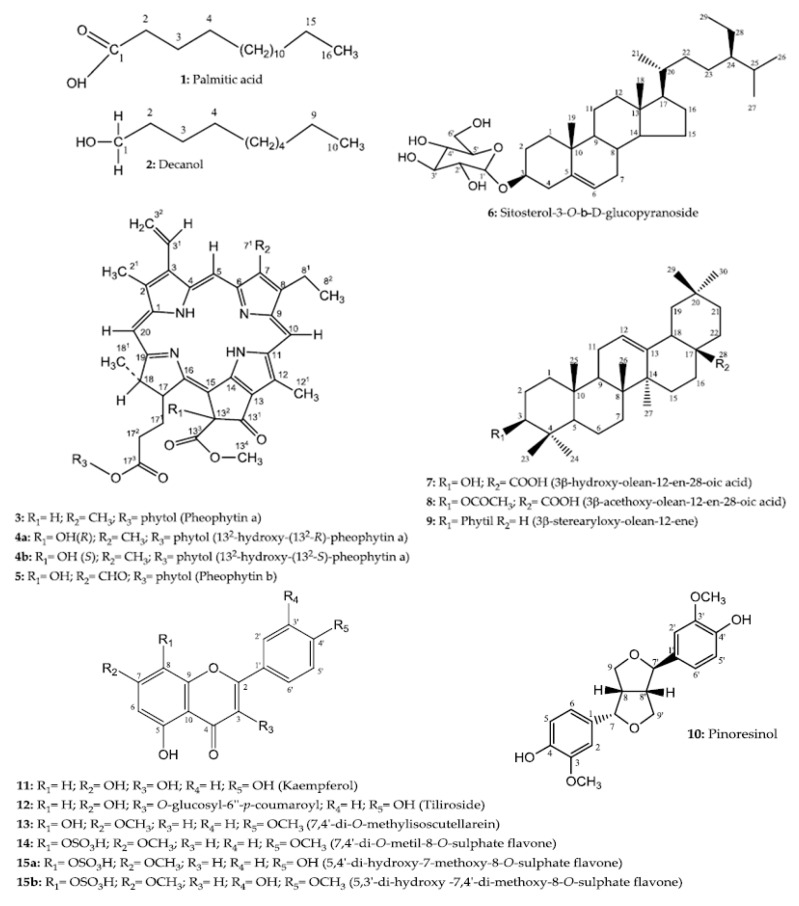
Compounds isolated from *H. velutina*.

The compounds identified from aerial parts of *H. velutina* are shown in Figure 1. They were identified by 1D and 2D NMR data and comparisons with the literature (spectra available as Supplementary material).

Fraction **15** was analyzed by NMR, IR and LC-HRMS. It was found to be a mixture of three compounds (**14**, **15a** and **15b**) and their spectral data are presented in Table 1.

#### Spectral Data

*Palmitic acid* (**1**), white solid; IR (KBr, cm^−1^): 3500, 2953, 2848, 1707, 1472, 1300. ^1^H-NMR (400 MHz) (CDCl_3_) δ: 2.34 (t, *J* = 7.4 Hz, 2H, H-2), 1.63 (q, 2H, H-15), 1.25 (bs), 0.87 (t, *J* = 6.6 Hz, H-16). ^13^C-NMR (δ, CDCl_3_, 100 MHz): 178.6 (C-1), 33.9 (C-2), 32.0 (C-4), 29.8 (C-5, C-12, C-13, C-14), 29.5 (C-11), 29.7 (C-6), 29.5 (C-7), 29.4 (C-8), 29.2 (C-9, C-10), 24.8 (C-3), 22.8 (C-15), 14.2 (C-16). The ^1^H- and ^13^C-NMR spectral data were consistent with published data [12].

*Decanol* (**2**), white solid; IR (KBr, cm^−1^): 3500, 2954, 2848, 1472. ^1^H-NMR (400 MHz) (CDCl_3_) δ: 3.63 (t, *J* = 6.56 Hz, 2H, 1H), 1.25–1.59 (m), 0.87 (t, *J* = 6.72 Hz, 3H, H-10). ^13^C-NMR (100 MHz) (CDCl_3_) δ: 63.0 (C-1), 32.7 (C-2), 29.3 (C-4), 29.4 (C-5), 29.6, 29.7, 31.9, 25.7 (C-3), 22.6 and 14.1 (C-10). The ^1^H- and ^13^C-NMR data were in accordance with published data [12].

Pheophytin a (**3**), 13^2^-hydroxy-(13^2^-*S*)-pheophytin a (**4a**), 13^2^-hydroxy-(13^2^-*R*)-pheophytin a (**4b**), Pheophytin b (**5**), green solids, were identified by 1D and 2D NMR and comparison with the literature [12,13,14].

*Sitosterol-3-O-β-d-glucopyranoside* (**6**), colorless crystal, was identified by 1D and 2D NMR and comparison with the literature [15].

*3-β-hydroxy-olean-12-en-28-oic acid* (**7**), white powder; ^1^H-NMR (500 MHz) (C_5_D_5_N) δ: 0.99 (s, 3H, Me-26), 1.22 (s, 3H, Me-24), 0.88 (s, 3H, Me-29), 0.93 (s, 3H, Me-25), 1.01 (s, 3H, Me-30), 1.01 (s, 3H, Me-23), 1.26 (s, 3H, Me-27), 3.29 (dd, 1H, H-18), 3.43 (dd, 1H, *J* = 5.6 e 10. 5 Hz, H-3), 5.48 (dd, 1H, *J* = 3.4 Hz, H-12); ^13^C-NMR (125 MHz) (C_5_D_5_N) δ: 16.5 (C-24), 15.5 (C-25), 17.4 (C-26), 18.7 (C-6), 23.6 (C-11), 23.7 (C-16), 23.7 (C-30), 26.1 (C-27), 28.0 (C-2), 28.7 (C-23), 30.9 (C-20), 33.1 (C-22), 33.1 (C-29), 33.2 (C-7), 34.1 (C-21), 37.7 (C-10), 38.8 (C-1), 39.3 (C-4), 39.7 (C-8), 41.9 (C-18), 42.1 (C-14), 46.4 (C-19), 48.0 (C-9), 55.7 (C-5), 78.0 (C-3), 122.5 (C-12), 144.8 (C-13), 180.2 (C-28). The ^1^H- and ^13^C-NMR spectral data were consistent with published data [16].

*3-β-acethoxy-olean-12-en-28-oic acid* (**8**), white powder; ^1^H-NMR (400 MHz) (CDCl_3_) δ: 0.73 (s, 3H, Me-26), 0.85 (s, 3H, Me-24), 0.91 (s, 3H, Me-29), 0.89 (s, 3H, Me-25), 0.93 (s, 3H, Me-30), 0.84 (s, 3H, Me-23), 1.11 (s, 3H, Me-27), 2.80 (dd, 1H, H-18), 4.49 (t, 1H, *J* = 8.5 Hz, H-3), 5.25 (dd, 1H, *J* = 3.4 Hz, H-12); 2.03 (s, 3H, OAc); ^13^C-NMR (δ, CDCl_3_, 100 MHz): 16.7 (C-24), 15.5 (C-25), 17.2 (C-26), 18.2 (C-6), 22.9 (C-11), 23.6 (C-16), 23.7 (C-30), 26.0 (C-27), 23.5 (C-2), 28.1 (C-23), 30.7 (C-20), 32.5 (C-22), 33.1 (C-29), 32.6 (C-7), 33.9 (C-21), 37.8 (C-10), 38.1 (C-1), 37.8 (C-4), 39.3 (C-8), 41.7 (C-18), 41.7 (C-14), 45.9 (C-19), 47.6 (C-9), 55.4 (C-5), 81.0 (C-3), 122.7 (C-12), 143.7 (C-13), 184.6 (C-28), 171.2 (OAc). The ^1^H-and ^13^C-NMR spectral data were consistent with published data [17].

*3-β-sterearyloxy-olean-12-ene* (**9**), white powder; ^1^H-NMR (400 MHz) (CDCl_3_) δ: 0.86 (s, 6H, Me-28, 18′), 0.87 (s, 6H, Me-23, 24), 0.95 (s, 3H, Me-25), 1.10 (s, 3H, Me-26), 0.96 (s, 3H, Me-27), 0.83 (s, 6H, Me-29, 30), 1,63 (m, H-3′), 2.30 (d, 2H, *J* = 7.8 Hz, H-2′), 1.25 (s, H-4′ a 17′) 4.59 (dd, 1H, *J* = 4.2, 7.3 Hz, H-3), 5.23 (t, *J* = 3.6 Hz, H-12); ^13^C-NMR (δ, CDCl_3_, 100 MHz): 16.9 (C-24), 15.6 (C-25), 17.2 (C-26), 18.5 (C-6), 23.9 (C-11), 27.1 (C-16), 23.8 (C-30), 26.1 (C-27), 23.5 (C-2), 28.0 (C-23), 31.2 (C-20), 47.3 (C-22), 33.4 (C-29), 32.6 (C-7), 34.8 (C-21), 36.5 (C-10), 38.7 (C-1), 37.2 (C-4), 40.5 (C-8), 47.6 (C-18), 41.0 (C-14), 46.9 (C-19), 47.9 (C-9), 55.8 (C-5), 80.6 (C-3), 122.1 (C-12), 144.5 (C-13), 28.4 (C-28), 173.8 (C-1′), 35.0 (C-2′), 25.3 (C-3′), 29.3 (C-4′), 29.4 (C-5′), 29.5 (C-6′), 29.7 (C-7′), 29.8 (C-8′ a C-13′), 29.8 (C-14′), 29.6 (C-15′), 32.0 (C-16′), 22.8 (C-17′), 12.2(C-18′). The ^1^H- and ^13^C-NMR spectral data were consistent with published data [18].

*Pinoresinol* (**10**), yellowish oil; ^1^H-NMR (400 MHz) (CD_3_COD_3_) δ_H_: 6.98 (d, *J* = 1.9 Hz, 2H, H-2, 2′), 6.78 (d, *J* = 8.1 Hz, 2H, H-5, 5′) 6.83 (dd, *J* = 8.1 e 2 Hz, 2H, H-6, 6′), 4.66 (d, *J* = 4.3 Hz, 2H, H-7, 7′), 3.08 (m, 2H, H-8, 8′), 4.20 (dd, *J* = 9 Hz, 2H, H-9, 9′), 3.78 (dd, *J* = 9.1 Hz, 2H, H-9, 9′), 3.84 (s, 6H, OMe-3, 3′); ^13^C-NMR (δ, CD_3_COD_3_, 100 MHz): 134.1 (C-1, 1′), 110.6 (C-2, 2′), 148.4 (C-3, 3′), 146.8 (C-4, 4′), 115.5 (C-5, 5′), 119.7 (C-6, 6′), 86.7 (C-7, 7′), 55.3 (C-8, 8′), 72.3 (C-9, 9′), 56.2 (C-3, 3′). The ^1^H- and ^13^C-NMR spectral data were in agreement with literature data [19]. 

*Kaempferol* (**11**), yellow powder: ^1^H-NMR (500 MHz) (CD_3_OD), δ_H_: 6.17 (d, *J* = 2.0 Hz, 1H), 6.39 (d, *J* = 2.0 Hz, 1H), 8.09 (d, *J* = 9.0 Hz, 2H, H-2′, 6′), 6.90 (d, *J* = 9.0 Hz, 2H, H-3′, 5′); ^13^C-NMR (125 MHz, CD_3_OD): 148.2 (C-2), 137.3 (C-3), 177.5 (C-4), 162.7 (C-5), 99.4 (C-6), 165.7 (C-7), 94.6 (C-8), 158.4 (C-9), 104.7 (C-10), 123.9 (C-1′), 130.9 (C-2′-6′), 115.7 (C-3′-5′), 160.7 (C-4′). The ^1^H- and ^13^C-NMR spectral data were in agreement with literature data [20].

*Tiliroside* (**12**), yellow powder; ^1^H-NMR (500 MHz) (DMSO-*d*_6_) δ_H_: 12.54 (s, 5-OH), 6.11 (*d*, *J* = 2.0 Hz, H-6), 6.35 (*d*, *J* = 2.0 Hz, H-8), 7.97 (*d*, *J* = 8.8 Hz, H-2′/6′), 6.84 (*d*, *J* = 8.8 Hz, H-3′/5′), 5.43 (*d*, *J* = 7.5 Hz, H-1′′), 3.14–3.26 (*m*, H-2′′, 3′′, 4′′, 5′′), 4,27 (dd, *J* = 2.0 e 12.0 Hz; 1H H-6″) e 4,02 (dd, *J* = 6.5 e 12.0 Hz 1H, H-6″) 7.35 (*d*, *J* = 8.5 Hz, H-2′′′/6′′′), 6.77 (*d*, *J* = 8.5 Hz, H-3′′′/5′′′), 7.33 (*d*, *J* = 15.5 Hz, H-7‴), 6.09(*d*, *J* = 16 Hz, H-8‴); ^13^C-NMR (125 MHz, DMSO-*d*_6_): 156.4 (C-2), 133.0 (C-3), 177.3 (C-4), 161.1 (C-5), 98.8 (C-6), 164.5 (C-7), 93.7 (C-8), 156.3 (C-9), 103.7 (C-10), 120.7 (C-1′), 130.1 (C-2′/6′), 115.7 (C-3′/5′), 159.9 (C-4′), 101.0 (C-1′′), 74.2 (C-2′′), 76.2 (C-3′′), 69.9 (C-4′′), 74.1 (C- 5′′), 62.9 (C-6′′), 124.9 (C-1′′′), 130.7 (2′′′, 6′′′), 115.0 (C-3′′′, 5′′′), 159.7 (C-4′′′), 144.5 (C-7′′′), 113..6 (C-8′′′), 166.1 (C-9′′′). The ^1^H- and ^13^C-NMR spectral data are were in agreement with literature data [21].

*7,4′-di-O-methyl isoscutellarein* (**13**), yellow powder; ^1^H-NMR (400 MHz) (DMSO-*d*_6_) δ_H_: 12.42 (s, 1H, 5-OH), 6.54 (*s*, 1H, Hz, H-6), 6.85 (*s*, 1H, Hz, H-3), 8.10 (*dd*, *J* = 2.1 and 6.8 Hz, 2H, H-2′, 6′), 7.12 (*dd*, *J* = 2.1 and 6.9 Hz, 2H, H-3′, 5′), 3.84 (s, 3H, OMe-4′), 3.89 (s, 3H, OMe-7′); ^13^C-NMR (100 MHz, DMSO-*d*_6_): 163.5 (C-2), 103.0 (C-3), 182.4 (C-4), 153.1 (C-5), 95.7 (C-6), 154.4 (C-7), 126.3 (C-8), 144.5 (C-9), 103.9 (C-10), 123.0 (C-1′), 128.5 (C-2′/6′), 114.6 (C-3′/5′), 163.5 (C-4′), 55.6 (OMe-4′), 56.4 (OMe-7). The NMR spectral data were in agreement with literature data [21].

*7,4′-di-O-methyl-8-O-sulphate flavone* (**14**), yellow powder; ^1^H-NMR (300 MHz δ_H_: 12.85 (s, 1H, 5-OH), 6.52 (*s*, 1H, Hz, H-6), 6.82 (*s*, 1H, Hz, H-3), 8.27 (*d*, *J* = 9.8 Hz, 2H, H-2′, 6′), 7.07 (*d*, *J* = 8.9 Hz, 2H, H-3′, 5′), 3.86 (s, 3H, OMe-4′), 3.85 (s, 3H, OMe-7′); ^13^C-NMR (75 MHz) (DMSO-*d*_6_): 164.3 (C-2), 102.7 (C-3), 182.1 (C-4), 156.9 (C-5), 96.0 (C-6), 159.1 (C-7), 122.7 (C-8), 149.4 (C-9), 103.7 (C-10), 123.1 (C-1′), 129.1 (C-2′/6′), 116.3 (C-3′/5′), 162.3 (C-4′), 55.5 (OMe-4′), 56.4 (OMe-7). The NMR spectral data were in agreement with literature data [21].

### 2.2. Biological Assay

**Figure 2:**
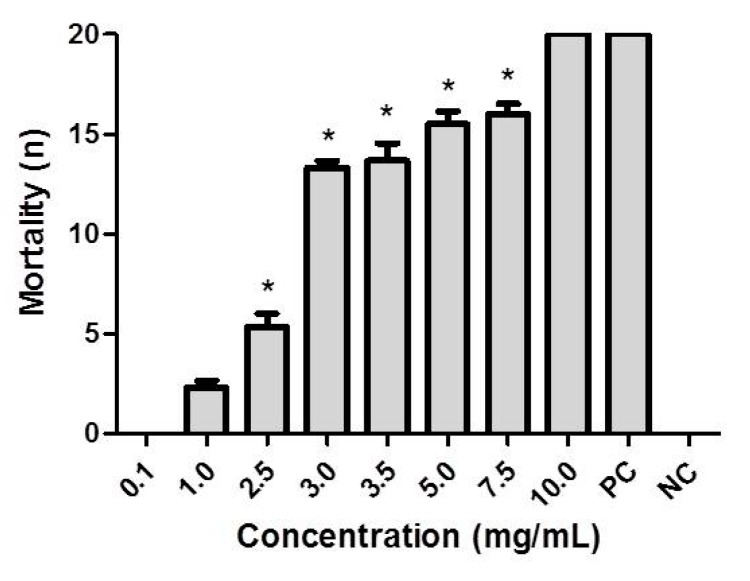
Larvicidal activity of different concentrations of Crude Ethanolic Extract (CEE) of *H. velutina* on *A. aegypti* larvae after 24 h. PC = Positive Control, NC = Negative Control. (*) Indicates results that are significantly different from controls.

The mean mortalities of *A. aegypti* larvae (L4) at each Crude Ethanolic Extract (CEE) concentration are depicted in Table 2. A concentration of 10.0 mg/mL was able to kill 100% of the larvae. Concentrations of 7.5, 5.0, 3.5, 3.0, 2.5, 1.0 and 0.1 mg/mL caused the death of 80.0%, 77.5%, 68.3%, 66.6%, 26.6, 11.6% and 0%, respectively. Only concentrations of 2.5 and 10.0 mg/mL were considered statistically different (*p* < 0.05), as can be seen in Figure 2. The calculated LC_50_ of CEE was 2.983 mg/mL.

## 3. Discussion

Sample **15** was obtained as a yellow powder. Its IR spectra recorded bands at 3466 cm^−1^, characteristic of hydroxyl axial deformation, and in the region of 2851 cm^−1^, typical of C-H from a methoxyl group [21]. It could be seen that there were absorptions at 1606, 1500 and 1450 cm^−1^, indicating a C=C of aromatic compounds, as well as at 1697 cm^−1^, suggestive of C=O of conjugated and bridged ketones present in flavonoids [22]. The absorbances of asymmetric stretches at 1384 cm^−1^ and symmetrical stretches at 1182 cm^−1^ indicated the occurrence of an S=O group, and together with absorptions at 1026 to 1001 cm^−1^, assigned to an S-O bond, pointed to the possible presence of a sulphate group in the structure [22].

The ^1^H-NMR spectra, obtained in DMSO-*d*_6_ exhibited a busy set of signals in the aromatic region, with different intensities suggesting that **15** might be a mixture of compounds. The signals of δ_H_ 8.26 (dd, *J* = 9.0 and 1.75 Hz, 2H) coupled with δ_H_ 7.09 (d, *J* = 9.0 Hz, 2H) and 8.13 (d, *J* = 8.9 Hz, 2H) with 6.86 (d, *J* = 8.8 Hz, 2H) suggested two AA′BB′ systems. The first system was indicative of a methoxyl substituent in C-4′, deshielding the 3′,5′ and 2′,6′ positions. The second system proposed the presence of an OH-4′ group, which protects H-3′,5′ and H-2′,6′. The substituents were later confirmed by 2D NMR analysis. The additional presence in the ^1^H-NMR spectra of signals at δ_H_ 7.56 (d, *J* = 2.2 Hz, 1H), 7.06 (d, *J* = 8.5 Hz, 1H) and 7.83 (dd, *J* = 2.2 and 8.5 Hz, 1H), compatible with an ABX system [21], suggested the existence of a third molecule in the mixture (Table 1). The ^1^H-NMR and IR spectral data provided evidence for the presence of a mixture of three flavones, renamed compounds **14**, **15a** and **15b**, respectively. A singlet at δ_H_ 3.82, with an intensity for three methoxyls in a chemically and magnetically equivalent environment, was consistent with the presence of this group at C-7 of the flavone nucleus of the three molecules. The signal at δ_H_ 3.84 was attributed to the methoxyl group of C-4′ of an AA′BB′ system, and the singlet at δ_H_ 3.85 suggested the ABX system, with OCH_3_-4′ and OH-3′, later confirmed by 2D NMR [21].

The APT ^13^C-NMR spectrum revealed weak peaks and aligned with HMBC, HMQC and COSY spectra, thereby allowing identification of the substances and confirming the position of the substituents. The ^13^C-NMR data showed carbons with high intensity at δ_C_ 129.3/115.9, characteristic values for the 2′/6′ and 3′/5′ carbons of the *para*-substituted B ring of flavonoids assigned to the major compound. The ^13^C-NMR spectrum showed methoxyl carbons at δ_C_ 56.4, 56.4 and 55.7.

The HRMS of the compounds were obtained by LC-HRMS, confirming the *O*-sulphate group in **14**, **15a** and **15b**. The accurate mass for compound **14** (minor compound) as an [M−H]^−^ ion, found with a retention time (RT) of 4.90 min, was 393.0260 (C_16_H_14_O_9_S); for the major compound, **15a**, the [M−H]^−^ ion (RT: 3.63 min) was 379.0129 (C_16_H_13_O_9_S); and for compound **15b**, also as an [M−H]^−^ ion (RT: 4.07 min) the mass found to be 409.0236 (C_17_H_15_O_10_S). The obtained results confirmed the *O*-sulphate group in the three molecules of the mixture and contributed to their identification.

Compound **14** was identified as 7,4′-di-*O*-methyl-8-*O*-sulphate-isoscutelarein, previously reported from *Wissadula periplocifolia* [21] and *Sidastrum micranthum* [23], both belonging to the Malvaceae family and here reported in the Sterculiaceae family. Compound **15a** was identified as 5,4′–di-hydroxy-7-methoxy-8-*O*-sulphate flavone, or Mariahine (named in honor of the author’s mother) and compound **15b** was identified as 5,3′–di-hydroxy-7,4′-dimethoxy-8-*O*-sulphate flavone, named as Condadine (in honor of the author’s hometown). Compounds **15a** and **15b** are being reported here for the first time in the literature.

Flavonoids with O-sulphated groups attached to the main skeleton are probably the most uncommon flavonoid derivatives and are found in few vegetal families. This is the first report of sulphated flavonoids in the Sterculiaceae family. These compounds are produced by cytosolic sulphotransferase (SOT) enzymes able to produce sulphated flavonoids as well as other sulphated metabolites. Different types of SOTs exist in the Golgi apparatus, where their role is to attach sulphate to protein and sugar structures [24].

The bioassays were performed using the concentration of 0.1 mg/mL, where no activity was observed. The concentration was gradually increased to reach a satisfactory mortal concentration. The larvae (L4) presented with compromised mobility and lethargy, followed by complete paralysis. This result became more intense when the CEE concentration was raised. Similar results have been described by other studies with the species *Swinglea glutinosa* [25], *Copaifera reticulata* and *Copaifera langsdorfii* (Leguminosae) [26,27].

According to Tukey’s testing, the concentrations 0.1 and 1.0 mg/mL did not differ significantly, and neither did the concentrations 3.0, 3.5, 3.5, 5.0 or 7.5 mg/mL when compared to 5.0 mg/mL and 7.5 mg/mL (Figure 2). This similarity of results from tested concentrations has already been reported in other studies [28,29,30,31]. The larvicidal percentage reached 100% after 24 h of exposure and the dose of 10.0 mg/mL was significantly more effective versus the other concentrations and negative control group.

According to the statistical analysis, the LC_50_ for the *H. velutina* aerial parts CEE was 2.983 mg/mL. The closest concentration tested was 3.0 mg/mL which killed 13.3 larvae (mean) corresponding to 66.6% of larvae. This concentration was much lower than those reported in previous studies evaluating larvicidal activity of extracts, such as *Croton linearifolius* (Euphorbiaceae), which presented an LC_50_ value of 17.420 mg/mL [29], and *Trichilia pallida* (Meliaceae) with an LC_50_ of 4.660 mg/mL [32]. The dose found was higher when compared to the *Duguetia furfuraceae* (Anonaceae) dose of 597 mg/mL [33] and the *Vitex gardneriana* (Verbenaceae) dose of 369 mg/mL [34].

The LC_10_, LC_50_ and LC_90_ calculated herein from the CEE of the aerial parts of *H. velutina* were more promising than the results from an earlier study [11], in which extracts of the stem and roots of the same species were evaluated (Table 3). This study shows that the aerial parts have significantly more larvicidal activity, arousing interest in evaluating the larvicidal activity of its constituents as well as the mechanisms of action involved. This difference in activity, depending on the part of the plant used, is common [35]; as exemplified by the larvicidal activity against *A. aegypti*, of the ethanolic extract of the leaves or roots of *Piper alatabaccum* (Piperaceae), with the leaves LC_50_ found as 869 mg/mL, while the LC_50_ value for the roots was 33 mg/mL. A study with *Azadirachta indica* (Meliaceae) showed there was a greater larvicidal potential for the ethanol extract of leaves (LC_50_ = 50 mg/mL) when compared with the root extract (LC_50_ = 600 mg/mL) [36].

Bioactive plant extracts usually present a synergistic or additive action by their compounds, being necessary for the subsequent evaluation of fractions and isolated compounds in order to determine if the complex matrix or the isolated compounds are more efficacious as larvicides [37]. Undoubtedly, the larvicidal activity of the *H. velutina* CEE in low concentrations justifies interest in using it as a domestic larvicide to combat *A. Aegypti* [37], a vector insect of severe viral diseases, such as dengue and Zika.

## 4. Materials and Methods

### 4.1. General

For the isolation and analysis of the compounds, the adsorbents Silica gel 60 (Merck), silica flash and/or Sephadex LH-20 (Merck, Kenilworth, NJ, USA) were used.

Infrared spectral data were obtained with a Perkin-Elmer FT-IR-1750 (Perkin-Elmer, São Paulo, SP, Brazil) using 1.0 mg of sample in KBr pellets measured in cm^−1^.

Nuclear magnetic resonance spectra were obtained using the spectrometers VARIAN-SYSTEM (Palo Alto, CA, USA) 500 MHz (^1^H) and 125 MHz (^13^C) , BRUKER 500 (Bruker, Coventry, UK) MHz (^1^H) and 100 MHz (^13^C) at Multiuser Laboratory Center of Characterization and Analysis (LMCA-UFPB) and VARIAN-GEMINI 300 MHz (^1^H) and 75 MHz (^13^C) at the *Centro Nordestino de Aplicação e Uso da Ressonância Magnética Nuclear* (CENAUREMN-UFC). Deuterated solvents were used in the dissolution of the samples for NMR. Chemical shifts (δ) were recorded in ppm (parts per million) and coupling constants (*J*) in Hz.

### 4.2. Collection, Extraction, and Compound Isolation

The aerial parts of *H. velutina* were collected in February 2015 in Serra Branca/Raso da Catarina (Jeremoabo City, Bahia, 09°53′15.5′′; 09°44′34.6′′ S and 38°49′36,1′′; 38°52′20.4′′ W) [38]. The material was identified by Prof. Adilva de Souza Conceição (UNEB) and a specimen voucher (28709-1) was kept in the Herbarium of the State University of Bahia (HUNEB, Paulo Afonso Collection).

The aerial parts of *H. velutina* were oven dried at 40 °C and 1976.0g of the powder was macerated with 95% ethanol (5 L) for 72 h. The extract solution was dried under reduced pressure at 40 °C and provided 39.7 g of CEE that was submitted to liquid-liquid chromatography using hexane, dichloromethane, ethyl acetate and n-butanol, resulting in their respective phases and a hydroalcoholic phase.

The hexane phase (11.0 g) was chromatographed in a silica gel column (CC), followed by medium pressure chromatography (MPC) with silica flash using hexane, ethyl acetate and methanol in increasing polarity mixtures. This process resulted in the isolation of substances **1** (7 mg), **2** (13 mg), **3** (30.0 mg), **4** (**4a** and **4b**—30.0 mg), **5** (12.0 mg), **7** (17.0 mg), **8** (22.0 mg) and **9** (6.0 mg).

The dichloromethane phase (8.0 g) was chromatographed in a silica flash CC using petroleum ether, dichloromethane and methanol in increasing polarity mixtures. The resulting fractions were analyzed and combined by similarity on TLC. Fractions **24**/**30** (815 mg) were chromatographed in flash silica CC with an elution system composed of hexane, ethyl acetate and methanol. The procedure resulted in the isolation of compounds **10** (8 mg) and **11** (6 mg).

The polar fractions were chromatographed in Sephadex (LH-20) CC employing, as the mobile phase, methanol and methanol:chloroform (1:1), providing compounds **6** (11 mg), **12** (85 mg), **13** (32 mg), **14** (37 mg) and **15** (**14**, **15a** and **15b)** (15 mg).

LC-MS (Accela 600 HPLC system combined with an Exactive (Orbitrap)—Thermo Fisher Scientific (Bremen, Germany)) was used to obtain the high-resolution mass spectra in negative or positive mode. The samples were solubilized in methanol (HPLC grade) to obtain a concentration of 1 mg/mL. The injection volume was 20 µL. The column used was a reverse phase ACE C-18 (150 × 3 mm, 3 µm) from HiChrom (Reading, UK). The mobile phase gradient was a mixture of 0.1% formic acid in H_2_O (solvent A) and acetonitrile (solvent B). The flow rate was 300 µL/min. The method is summarized in Table 4. The obtained results were analyzed using Xcalibur 2.2 (Thermo Fisher Scientific) (Bremen, Germany).

### 4.3. Biological Assay

The larvicidal activity of the CEE of *H. velutina* was evaluated following the recommendations of the World Health Organization (1970). The fourth-stage *A. aegypti* larvae (L4) (Rockefeller strain) were obtained from the Laboratory of Biotechnology Applied to Parasites and Vectors, Biotechnology Center, Federal University of Paraiba.

*H. velutina* CEE was diluted in distilled water (10 mL) at different concentrations (0.1 to 10 mg/mL). Twenty L4-stage larvae were transferred into Falcon tubes containing the solutions of *H. velutina* CEE. One control group was prepared using only water. The positive control group was prepared using a solution of the insecticides Imiprothrin 0.02%, Permethrin 0.05% and Esbiothrin 0.1%. The tubes were incubated for 24 h at 28 ± 4 °C, over 12 h of natural light and 12 h of darkness. Larvae mortality was verified after 24 h of incubation. All tests were carried out in triplicate. GraphPad Prism 5.0 software (GraphPad Software, San Diego, CA, USA) was used to calculate LC_10_, LC_50_ and LC_90_. Analysis of variance (ANOVA) and Tukey’s test (*p* < 0.05) were applied to determine significant differences between groups.

## 5. Conclusions

The phytochemical study of the crude ethanolic extract of *H. velutina* aerial parts led to the identification of 16 compounds. Among them were one fatty acid, one aliphatic alcohol, four chlorophyll derivatives, one steroid, triterpenes, a lignan, and flavonoids, highlighting the novel sulphated flavonoids, 5,4′-di-hydroxy-7-methoxy-8-*O*-sulphate flavone (mariahine) (**15a**) and 5,3′-di-hydroxy-7,4′-methoxy-8-*O*-sulphate flavone (condadine) (**15b**). The present work contributed to consolidating the chemotaxonomic knowledge of the Sterculiaceae family, reporting for the first time the production of sulphated flavonoids in this family. The CEE of *H. velutina* aerial parts exhibited robust larvicidal activity against *A. aegypti* larvae, demonstrating that the extract can be useful for developing domestic larvicides to combat *A. Aegypti* [37], a vector insect of severe viral diseases, such as dengue and Zika.

## Figures and Tables

**Table 1:** NMR data (^1^H, ^13^C and HMBC- Heteronuclear Multiple Bond Correlation) of **14**, **15a** and **15b** (δ, DMSO-*d*_6_, 500 and 125 MHz).

C	14			15a			15b		
	δ_H_	δ_c_	HMBC	δ_H_	δ_C_	HMBC	δ_H_	δ_c_	HMBC
2	-	164.3	-	-	164.8	-	-	164.7	-
3	6.74 (s, 1H)	101.8	C-2	6.74 (s, 1H)	101.8	C-2, C-1′	6.69 (s, 1H)	102.7	-
4	-	182.6	-	-	182.6	-	-	182.6	-
5	-	-	-	-	-	-	-	-	-
6	6.48 (s, 1H)	95.8	-	6.48 (s, 1H)	95.8	-	6.48 (s, 1H)	95.8	
7	-	159.1		-	159.1		-	159.1	-
8	-	123.1	-	-	123.2	-	-	123.2	-
9	-	148.8	-	-	148.8	-	-	148.8	-
10	-	104.7	-	-	104.7	-	-	104.7	-
1′	-	123.6	-	-	121.6	-	-	123.6	-
2′	8.26 (d, *J* = 1.75 e 9.0 Hz, 2H)	129.3	C-2, C-4′, C-6′	8.13 (d, *J* = 8.9 Hz, 2H)	129.3	C-2, C-4′, C-6′	7.56 (d, *J* = 2.2 Hz 1H)	113.8	C-3′, C-4′, C-6′
3′	7.09 (d, *J* = 9.0 Hz, 2H)	116.2	C-4′, C-1′	6.86 (d, *J* = 8.8 Hz, 2H)	116.0	C-4′, C-1′, C-5′	-	146.8	-
4′	-	162.7	-	-	162.3	-	-	151.6	-
5′	7.09 (d, *J* = 9.0 Hz, 2H)	116.2	C-4′, C-1′	6.86 (d, *J* = 8.8 Hz, 2H)	116.0	C-4′, C-1′	7.07 (d, *J* = 8.5 Hz, 1H)	111.8	C-1′
6′	8.26 (d, *J* = 1.75 e 9.0 Hz, 2H)	129.3	C-2, C-4′	8.13 (d, *J* = 8.9 Hz, 2H)	129.3	C-2, C-4′	7.83 (dd, *J* = 2.2 e 8.5 Hz, 1H)	119.8	C-2′, C-4′
OCH_3_-4′	3.84 (s, 3H)	56.4	-	-	-	-	3.85 (s, 3H)	55.7	-
OCH_3_-7	3.82 (s, 3H)	56.4	-	3.82 (s, 3H)	56.4	-	3.82 (s, 3H)	56.4	
OH-5	12.91 (s, 1H)	-	-	12.91 (s, 1H)	-	-	12.91 (s, 1H)	-	-

**Table 2:** Mean number of mortalities of *A. aegypti* larvae (L4) in different concentrations of Crude Ethanolic Extract (CEE) of *H. velutina*.

Concentration (mg/mL)	Mean Mortality (n)	Standard Deviation (Triplicate)
0.1 (a) *	0	0
1.0 (a)	2.3 (11.6%)	0.57
2.5	5.3 (26.6)	1.15
3.0 (b) (c)	13.3 (66.6%)	0.57
3.5 (b) (d) (e)	13.6 (68.3%)	1.52
5.0 (c) (d) (f)	15.5 (77.5%)	1.29
7.5 (e) (f)	16.0 (80.0%)	1.26
10.0	20 (100%)	0
Negative Control	0	0
Positive Control	20 (100%)	0

* Means followed by the same letter are not significantly different by Tukey test, at a level of 5% of probability.

**Table 3:** Lethal concentrations (LC_10_, LC_50_ and LC_90_) of *Helicteres velutina* CEE against *Aedes aegypti* larvae (24 h of exposure) [11].

Used Part	LC_10_	LC_50_	LC_90_
Stem *	60.406 mg/mL	138.896 mg/mL	319.372 mg/mL
Roots *	73.029 mg/mL	171.683 mg/mL	403.607 mg/mL
Aerial parts	0.965 mg/mL	2.983 mg/mL	9.691 mg/mL

* Santos et al., 2012 [11].

**Table 4:** LC-HRMS gradient method.

Time (min)	A%	B%
0	75	25
15	25	75

## Supplementary Materials

The following are available online.
