# Supplementary material for: New Sulphated Flavonoids and Larvicidal Activity of Helicteres velutina K. Schum (Sterculiaceae)
Source: Molecules. 2018 Oct 27;23(11):2784. doi: 10.3390/molecules23112784 (PMC6278388; doi:10.3390/molecules23112784)
Supplement: Supplementary file 1 [file molecules-23-02784-s001.pdf]

New Sulphated Flavonoids and Larvicidal Activity of *Helicteres velutina* K. Schum (Sterculiaceae)

Diégina A. Fernandes [1], Maria S. R. Souza [1], Yanna C. F. Teles [2], Louise H. G. Oliveira [3], Jéssica B. Lima [4], Micaelly da S. Oliveira[5], Adilva S. Conceição [4], Fabíola C. Nunes [3], Tania M. S. Silva[6] and Maria de Fátima Vanderlei de Souza [1,5,*]

[1] Post graduation Program in Bioactive Natural and Synthetic Products; Federal University of Paraíba, 58051-900, João Pessoa, PB, Brazil; diegina@ltf.ufpb.br (D.A.F.); sallett@ltf.ufpb.br (M.S.R.S.)

[2] Department of Chemistry and Physics, Agrarian Sciences Center, Federal University of Paraíba, Areia, PB, Brazil; yanna@cca.ufpb.br (Y.C.F.T.)

[3] Biotechnology Center; Federal University of Paraíba, 58051-900, João Pessoa, PB, Brazil; louiseguimaraes@outlook.com (L.H.G.O.); fabiola@cbiotec.ufpb.br (F.C.N.)

[4] Post graduation Program in Plant Biodiversity; University of the State of Bahia; Department of Education, 41150-000, Paulo Afonso, BA, Brazil; jessica.bl@hotmail.com (J.B.L) ; adilva.souza@gmail.com (A.S.C.)

[5] Post graduation in Development and Technological Innovation in Medicines; Federal University of Paraíba, 58051-900, João Pessoa, PB, Brazil; mica_ellysilva@hotmail.com (M.S.O.); mfvanderlei@ltf.ufpb.br (M.F.V.S)

[6] Department of Molecular Sciences, Rural Federal University of Pernambuco, Campus Dois Irmãos, 52171-900, Recife, PE, Brazil; sarmentosilva@gmail.com (T.M.S.S.)

* Correspondence: mfvanderlei@ltf.ufpb.br; Tel.: +55-83-3216-7351; Fax: +55-83-3216-7351

Abstract: *Helicteres velutina* K. Schum (Sterculiaceae), commonly known in Brazil as 'pitó' is traditionally used by indigenous as insecticide and repellent. The present work reports on the phytoconstituents from aerial parts of *H. velutina* and evaluation of the larvicidal potential of its extract. The compounds were isolated by chromatography and identified by NMR, IR and LC-HRMS. The study led to the isolation of a fatty acid, one aliphatic alcohol, four chlorophyll derivatives, one steroid, triterpenes, a lignan, and flavonoids, highlighting the new compounds in the literature, 5,4'-di-hydroxy-7-methoxy-8-O-sulphate flavone (mariahine) (**15a**) and 5,3'–di-hydroxy-7,4'-dimethoxy-8-O-sulphate flavone (condadine) (**15b**). The present work contributes to the chemotaxonomic knowledge of the Sterculiaceae family, reporting for the first time the occurrence of sulphated flavonoids in this family. The crude ethanolic extract of *H. velutina* showed great larvicidal activity against *Aedes aegypti* larvae, showing that the extract can be useful as a domestic larvicide, as indicated by traditional use, to combat *Ae. aegypti*, a vector insect of severe virus diseases, such as *dengue* and *Zika*,

Keywords: *Helicteres velutina*; sterculiacaeae; sulphated flavonoids; larvicidal activity; *Aedes aegypti*

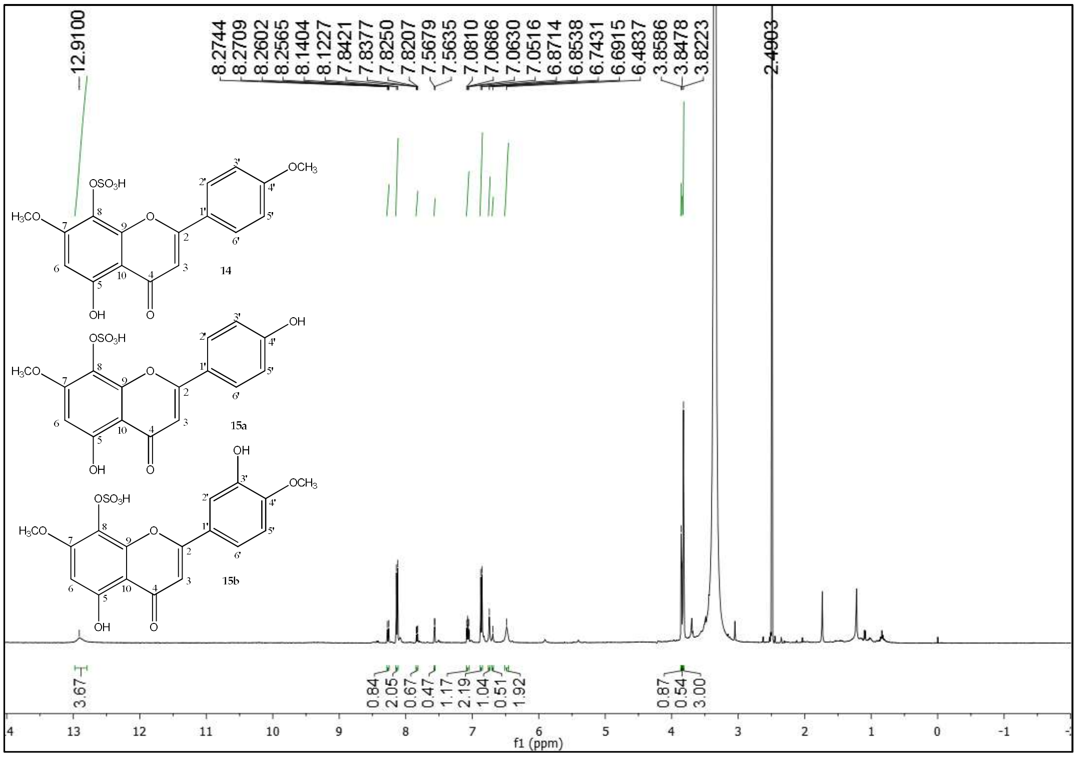

Figure S1. ^{1}H-NMR spectrum (500 MHz, DMSO) of **14 + 15a +15b**

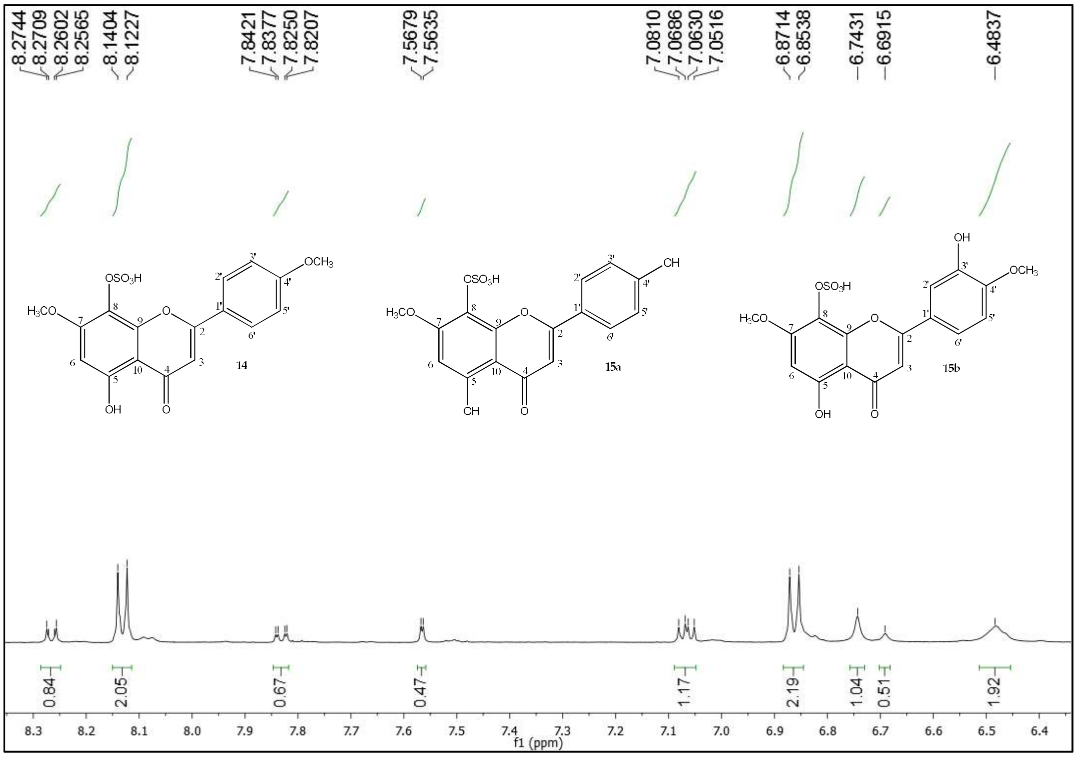

Figure S2. Expansion of ^{1}H-NMR spectrum (500 MHz, DMSO) of **14 + 15a +15b**.

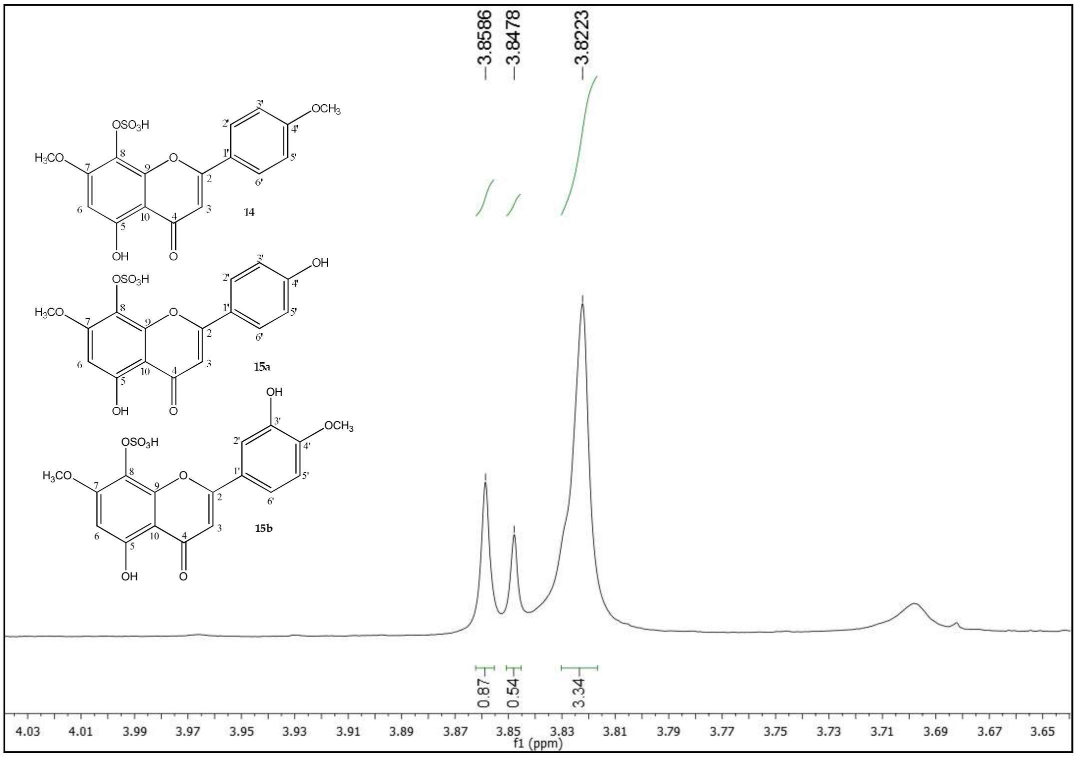

Figure S3. Expansion of ^{1}H-NMR spectrum (500 MHz, DMSO) of **14 + 15a +15b**.

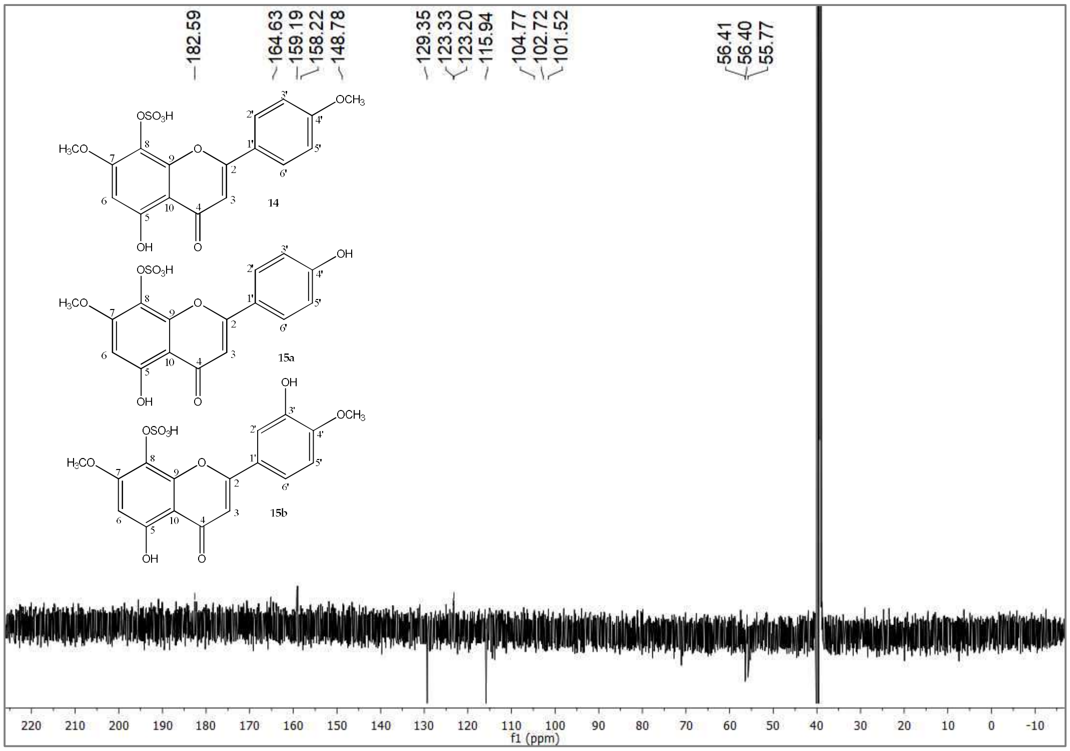

Figure S4. ^{13}C-NMR spectrum (125 MHz, DMSO) of **14 + 15a +15b**.

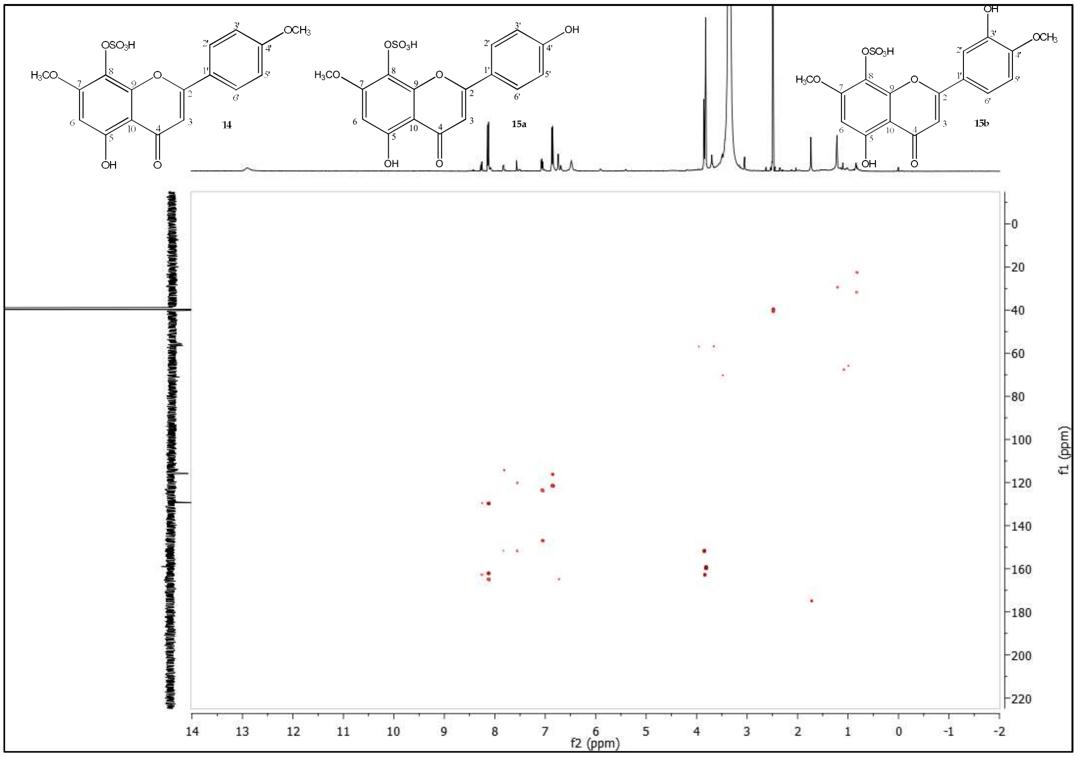

Figure S5. HMBC spectrum (^{1}H-NMR: 500 MHz, ^{13}C-NMR: 125 MHz, DMSO) of **14 + 15a +15b**.

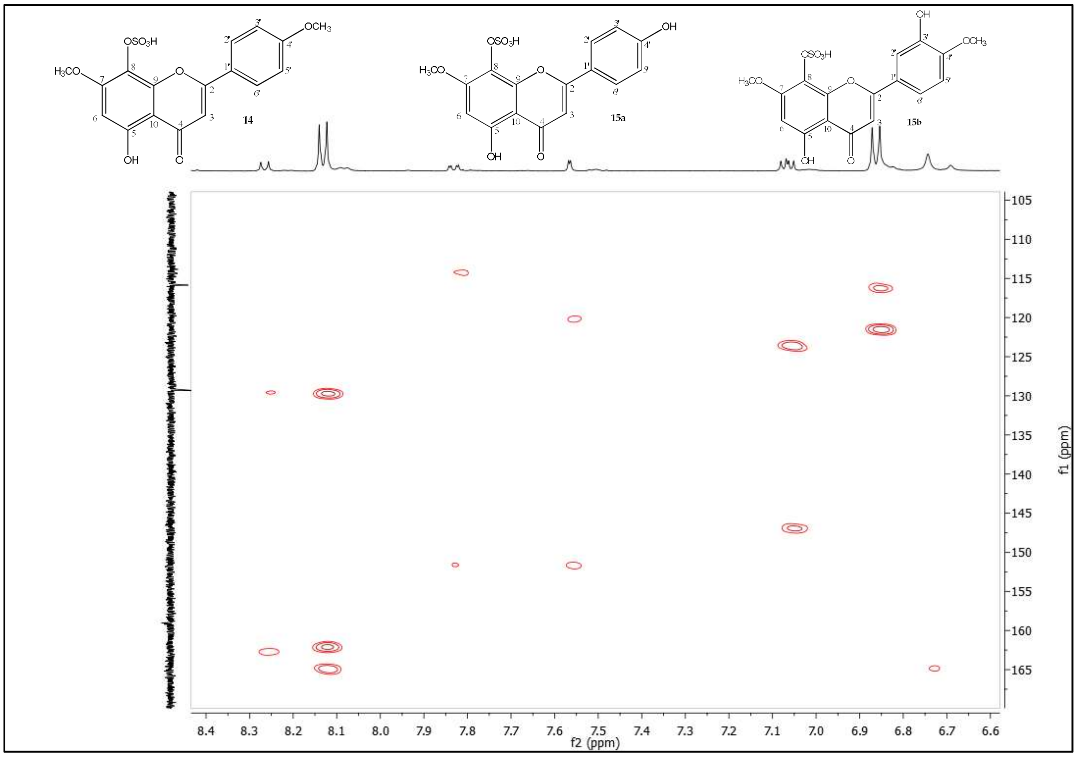

Figure S6. Expansion of HMBC spectrum (^{1}H-NMR: 500 MHz, ^{13}C-NMR: 125 MHz, DMSO) of **14 + 15a +15b**.

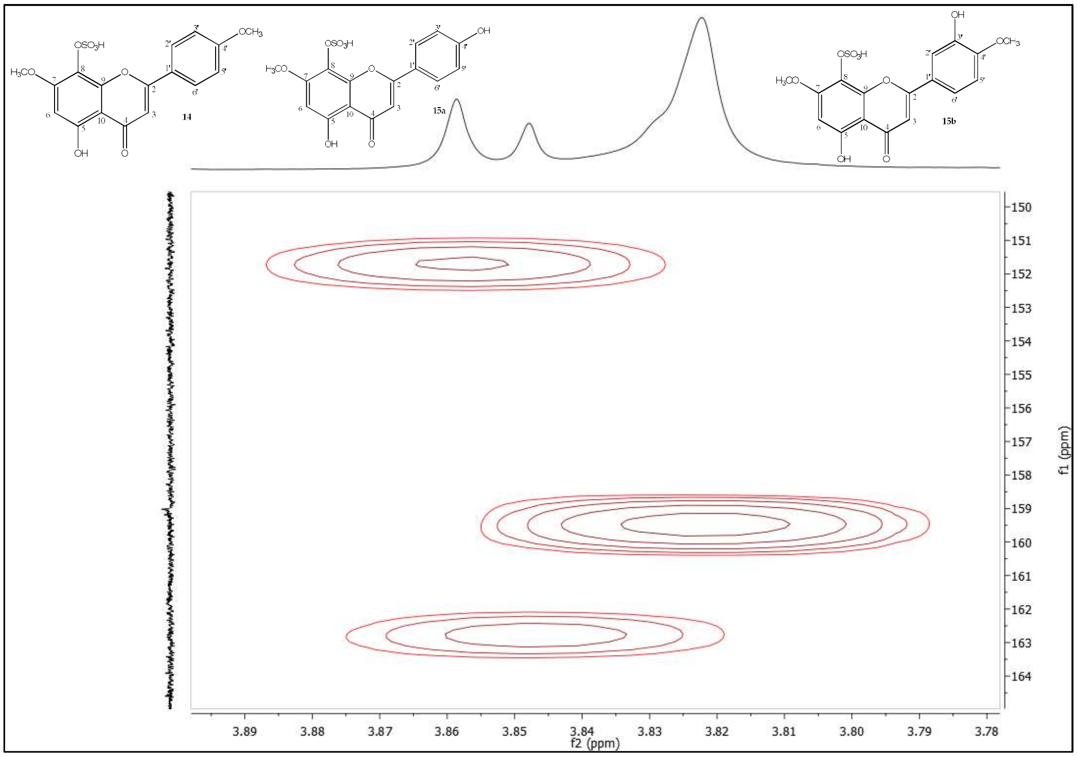

Figure S7. Expansion of HMBC spectrum (^{1}H-NMR: 500 MHz, ^{13}C-NMR: 125 MHz, DMSO) of **14 + 15a +15b**.

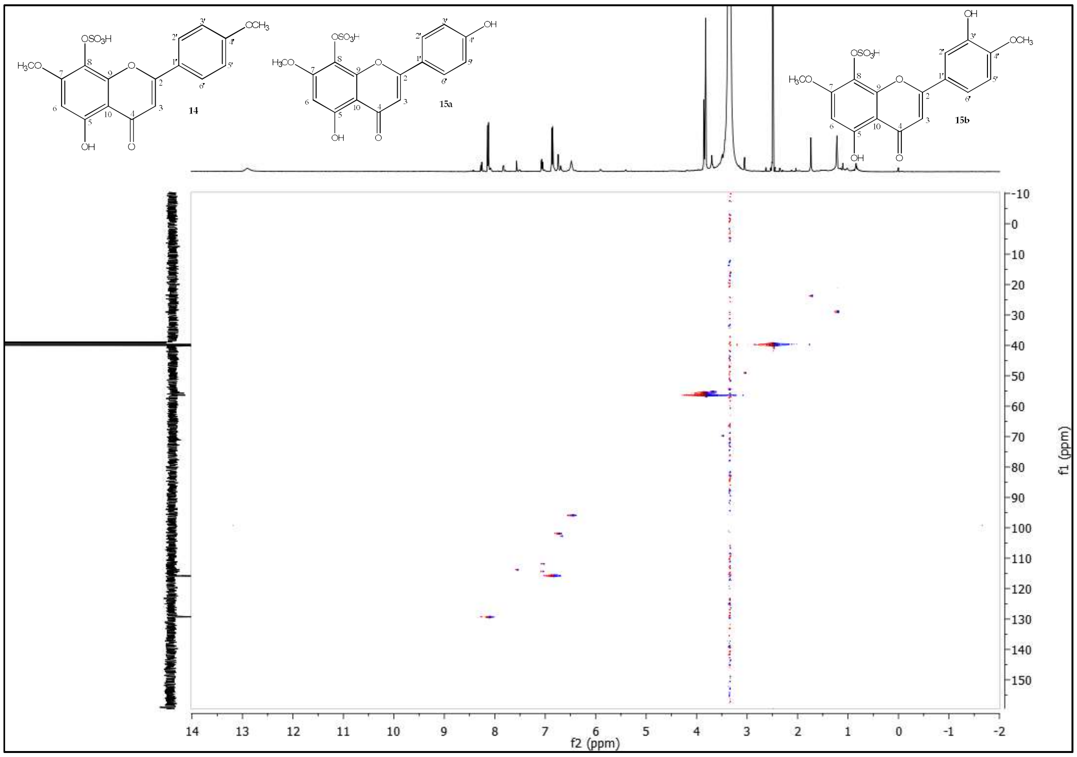

Figure S8. HMQC spectrum (^{1}H-NMR: 500 MHz, ^{13}C-NMR: 125 MHz, DMSO) of **14 + 15a +15b**.

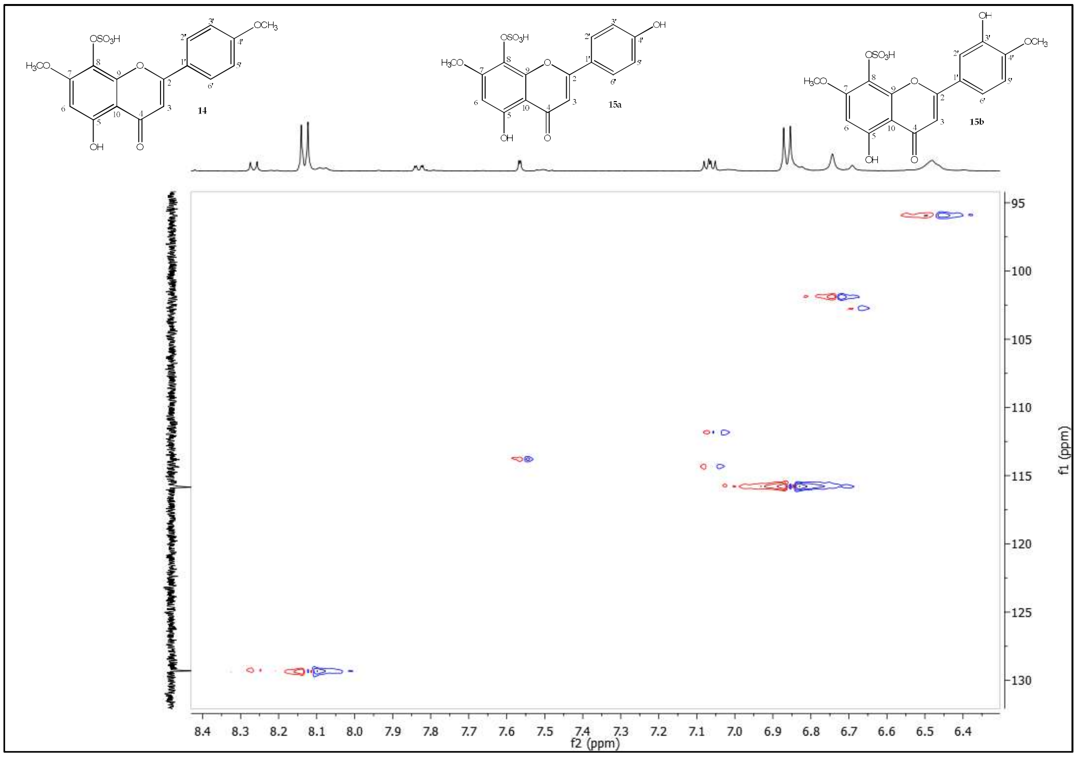

Figure S9. Expansion of HMQC spectrum (^{1}H-NMR: 500 MHz, ^{13}C-NMR: 125 MHz, DMSO) of **14 + 15a +15b**.

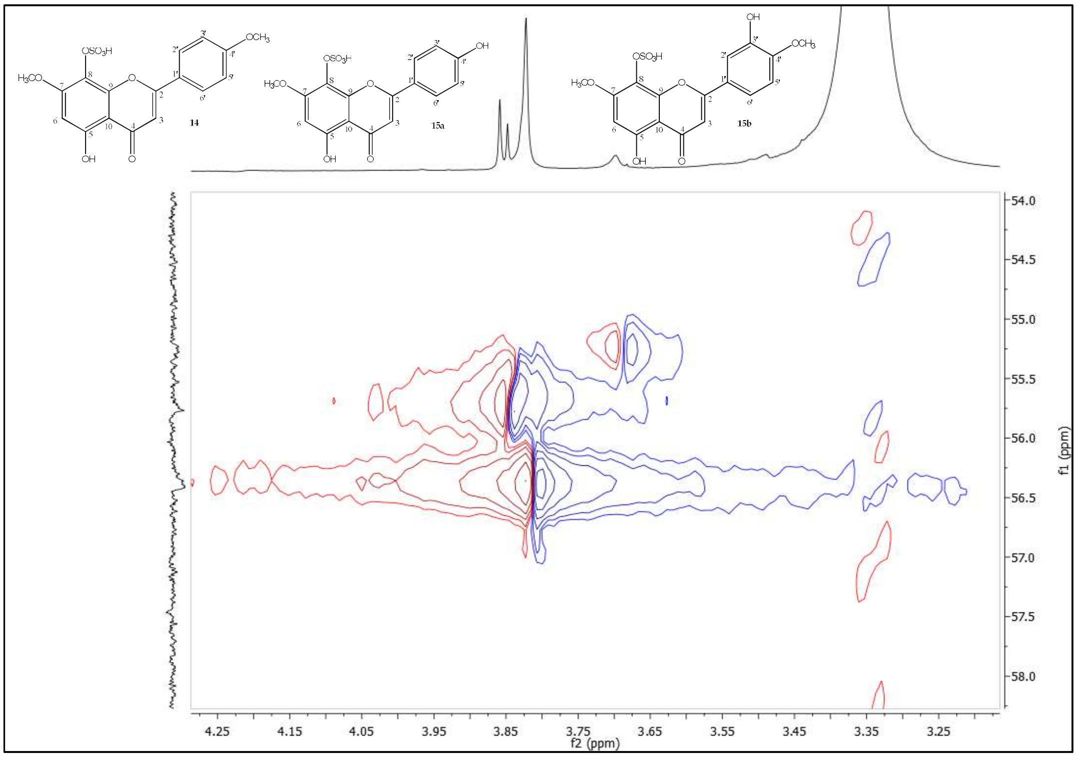

Figure S10. Expansion of HMQC spectrum (^{1}H-NMR: 500 MHz, ^{13}C-NMR: 125 MHz, DMSO) of **14 + 15a +15b**.

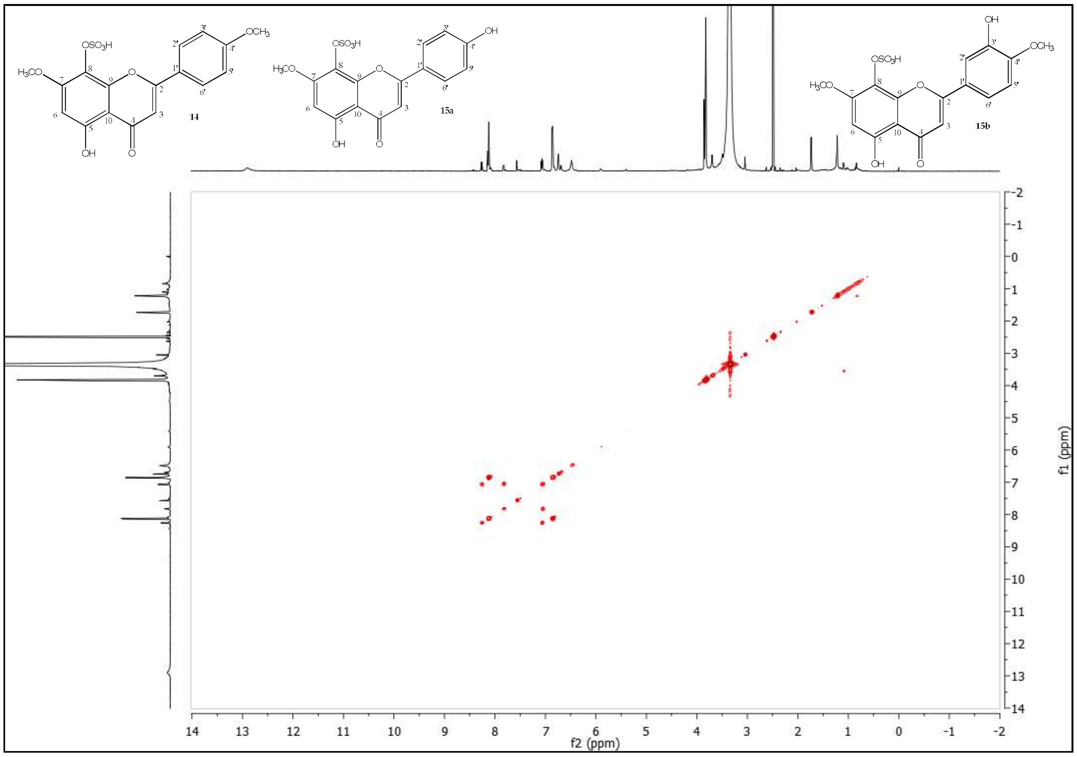

Figure S11. Cosy spectrum (^{1}H-NMR: 500 MHz, DMSO) of **14 + 15a +15b**.

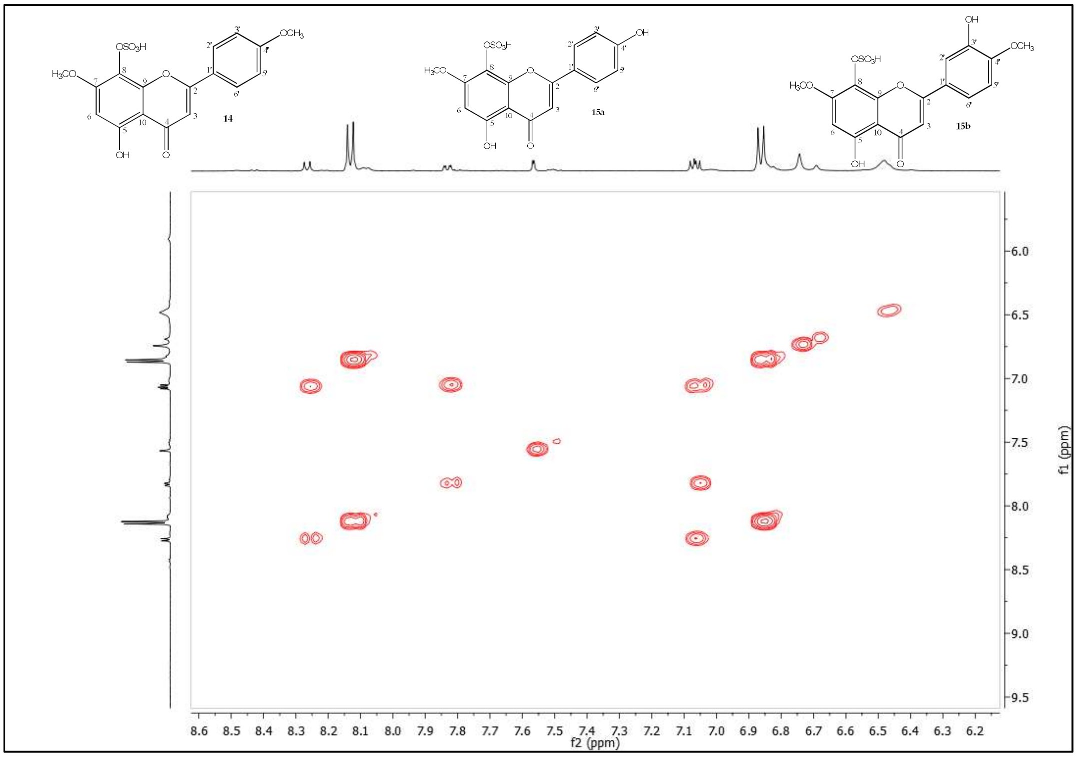

Figure S12. Expansion of COSY spectrum (^{1}H-NMR: 500 MHz, DMSO) of **14 + 15a +15b**.

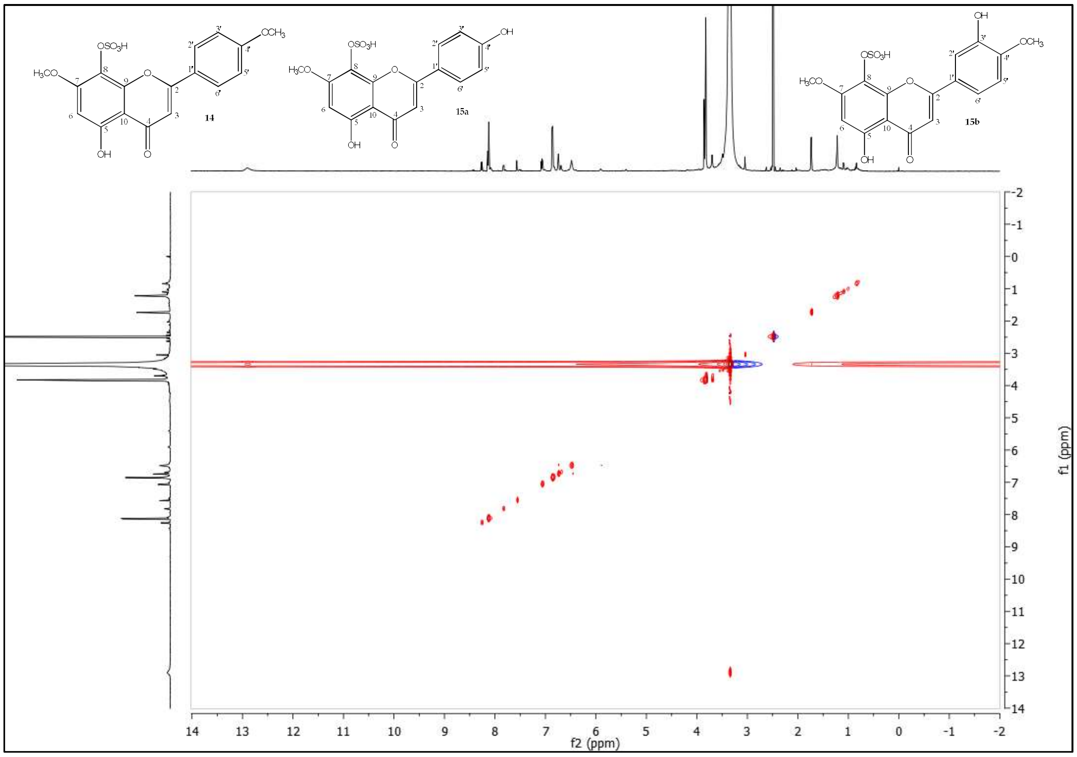

Figure S13. NOESY spectrum (^{1}H-NMR: 500 MHz, DMSO) of **14 + 15a +15b**.

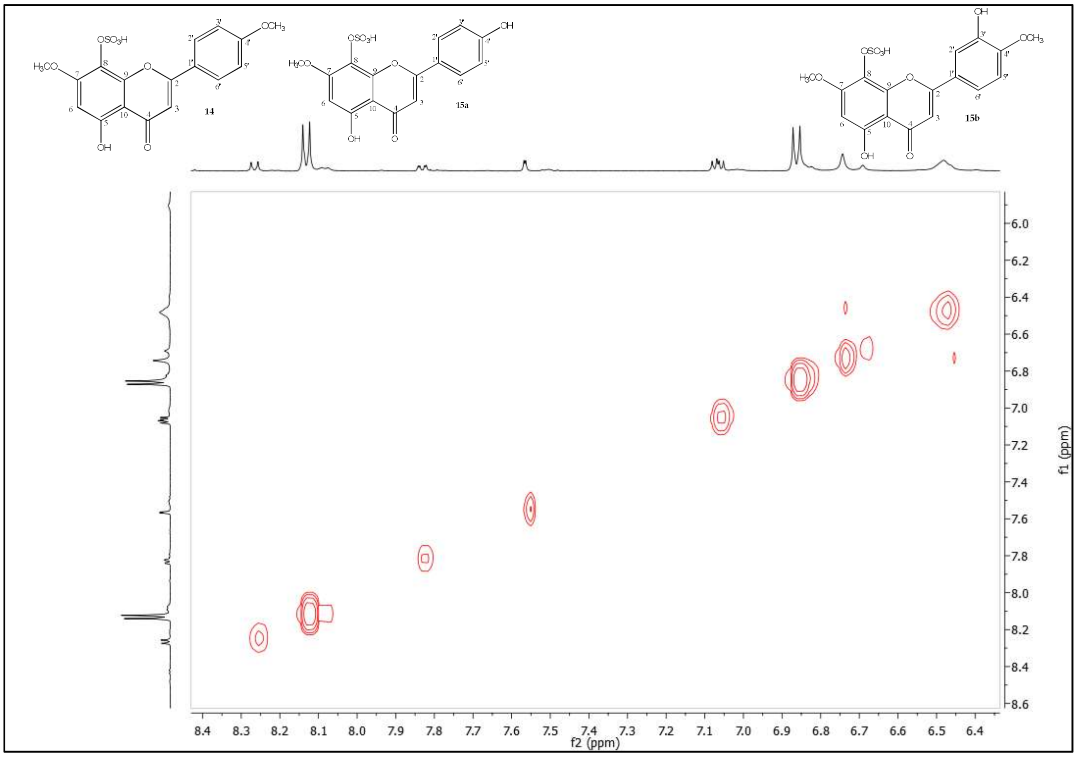

Figure S14. Expansion of NOESY spectrum (^{1}H-NMR: 500 MHz, DMSO) of **14 + 15a +15b**.

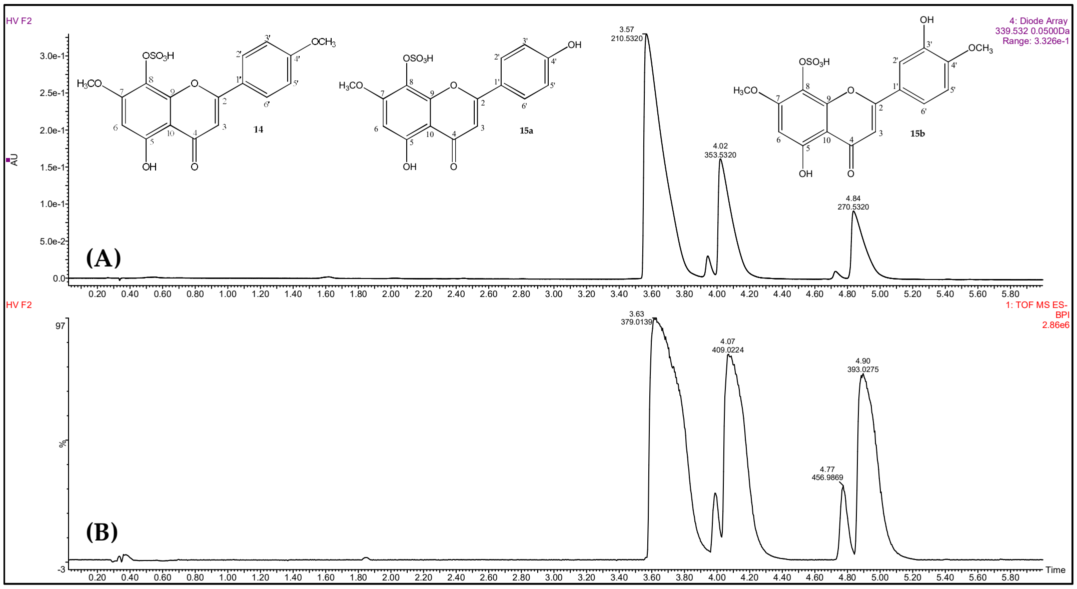

Figure S15. UV spectrum at 340 nm at UPLC-DAD (**A**) and ESI base peak ion (BPI) chromatogram of **14 + 15a +15b** mixture analyzed by UPLC-QTOF-MS (**B**).

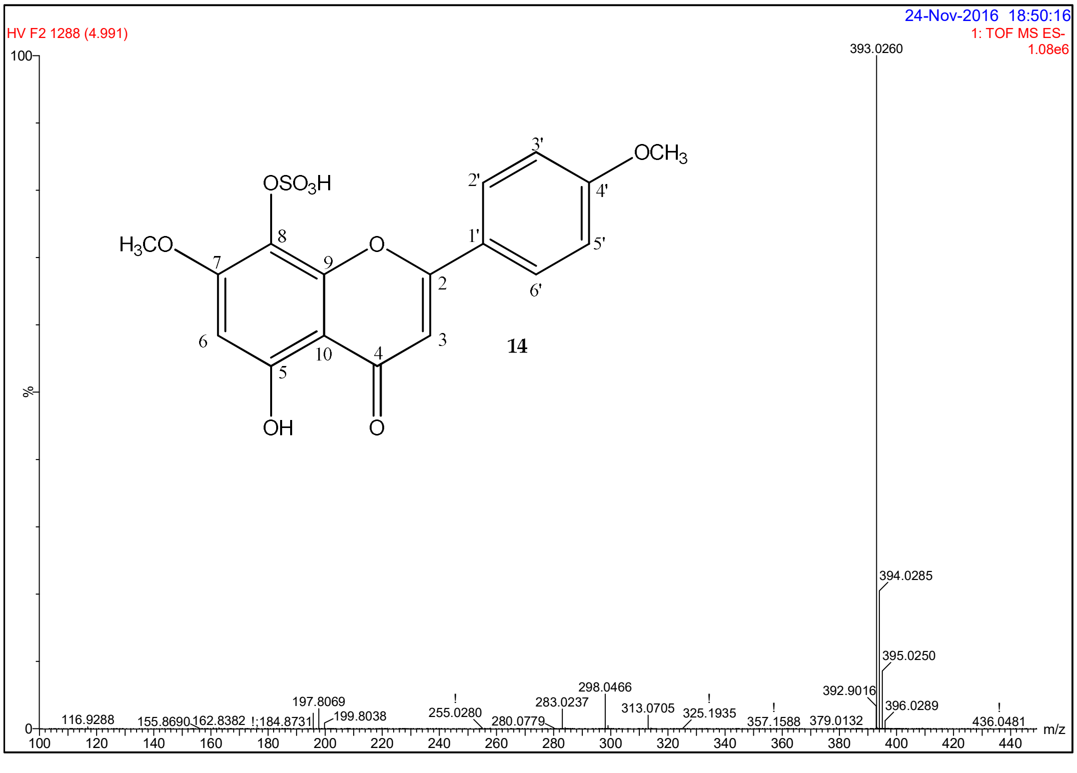

Figure S16. HRMS spectrum of compound **14**

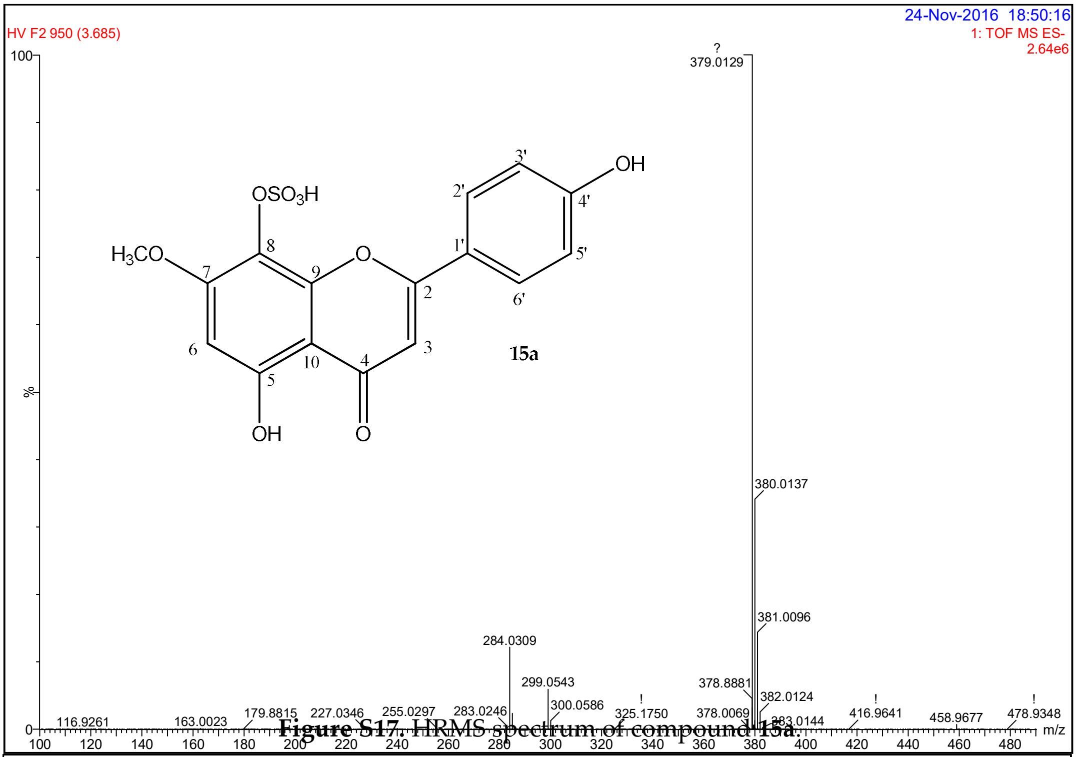

Figure S17. HRMS spectrum of compound **15a**.

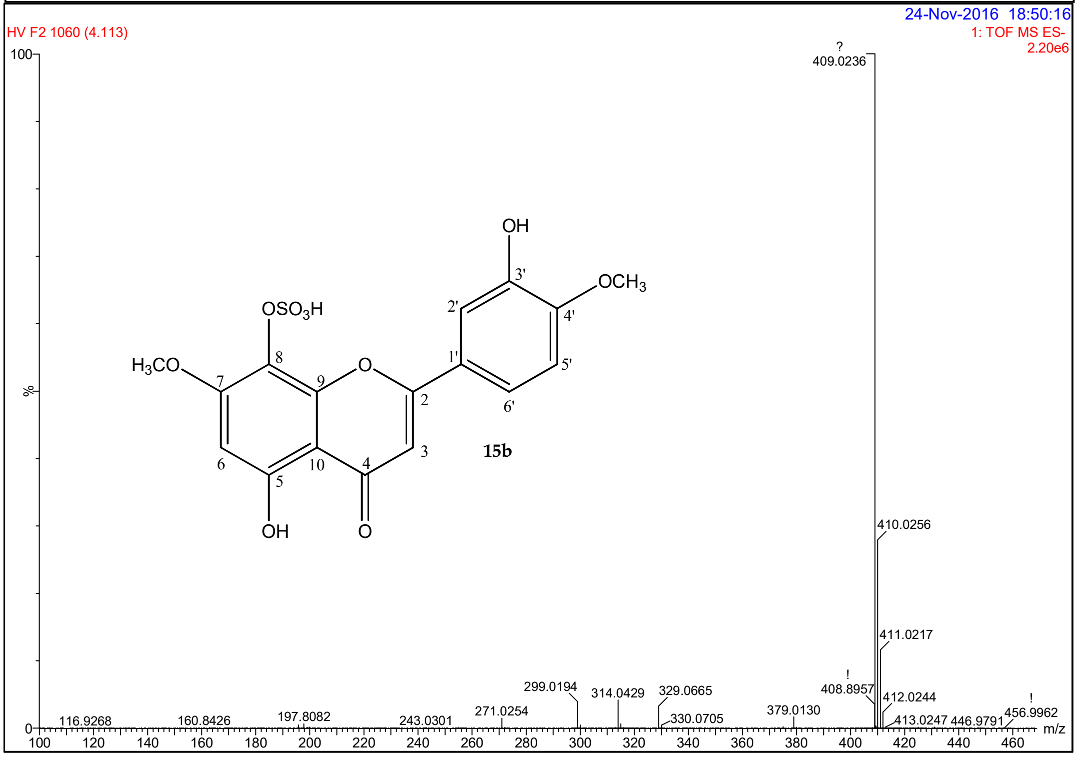

Figure S18. HRMS spectrum of compound **15b**.

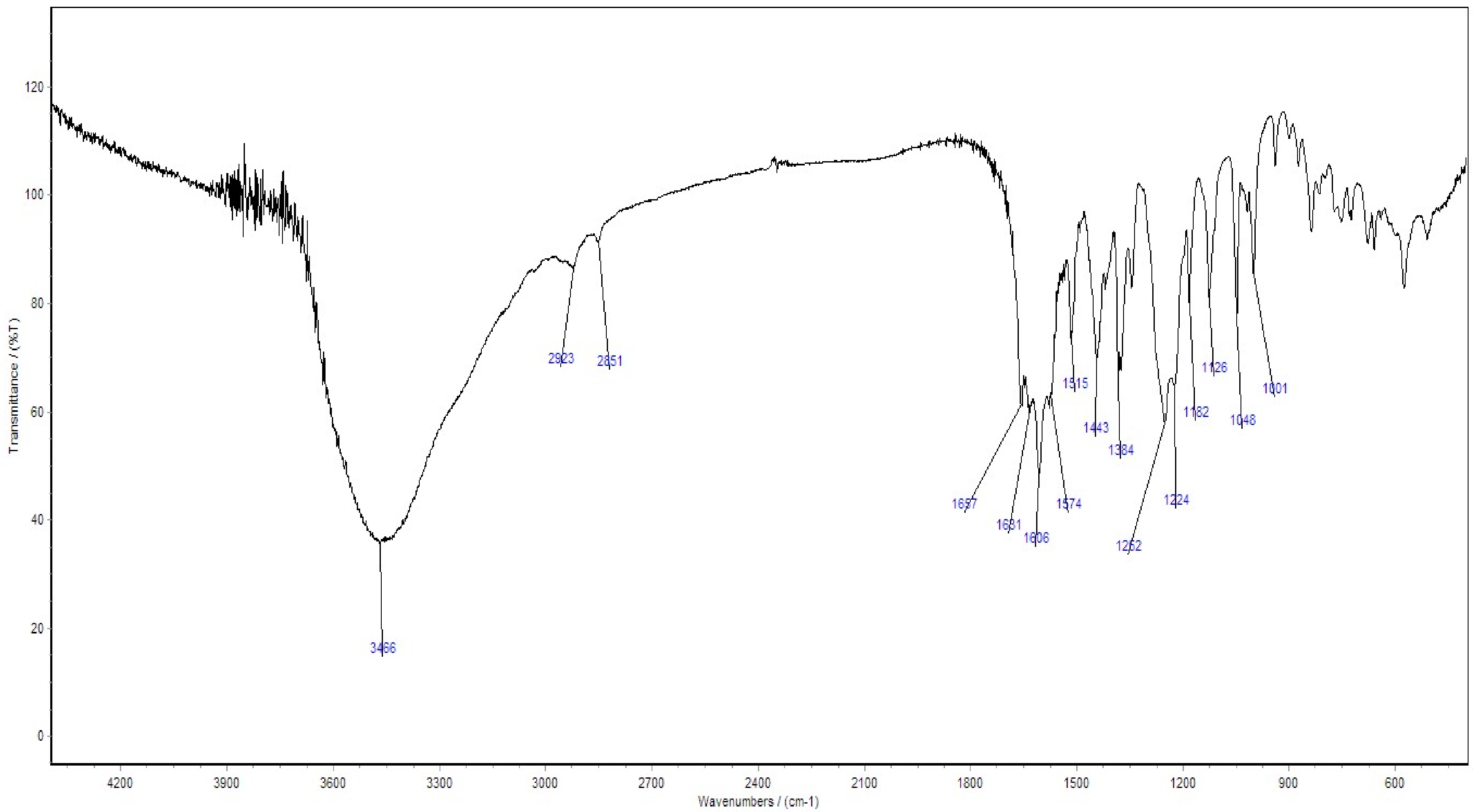

Figure S19. IV spectrum (cm^{-1}, KBr) of **14 + 15a +15b**.
